# Geographical Gradient of the *eIF4E* Alleles Conferring Resistance to Potyviruses in Pea (*Pisum*) Germplasm

**DOI:** 10.1371/journal.pone.0090394

**Published:** 2014-03-07

**Authors:** Eva Konečná, Dana Šafářová, Milan Navrátil, Pavel Hanáček, Clarice Coyne, Andrew Flavell, Margarita Vishnyakova, Mike Ambrose, Robert Redden, Petr Smýkal

**Affiliations:** 1 Department of Plant Biology, Mendel University in Brno, Brno, Czech Republic; 2 Department of Cell Biology and Genetics, Palacky University in Olomouc, Olomouc, Czech Republic; 3 CEITEC MENDELU, Mendel University in Brno, Brno, Czech Republic; 4 Western Regional Plant Introduction Station - USDA, Pullman, Washington, United States of America; 5 Division of Plant Sciences, University of Dundee at James Hutton Institute, Invergowrie, United Kingdom; 6 Vavilov Institute of Plant Industries, Saint Petersburg, Russian Federation; 7 John Innes Centre, Norwich, United Kingdom; 8 Australian Grains Genebank, Horsham, Victoria, Australia; 9 Department of Botany, Palacky University in Olomouc, Olomouc, Czech Republic; Instituto de Higiene e Medicina Tropical, Portugal

## Abstract

**Background:**

The eukaryotic translation initiation factor 4E was shown to be involved in resistance against several potyviruses in plants, including pea. We combined our knowledge of pea germplasm diversity with that of the *eIF4E* gene to identify novel genetic diversity.

**Methodology/Principal findings:**

Germplasm of 2803 pea accessions was screened for *eIF4E* intron 3 length polymorphism, resulting in the detection of four *eIF4E^A-B-C-S^* variants, whose distribution was geographically structured. The *eIF4E^A^* variant conferring resistance to the P1 PSbMV pathotype was found in 53 accessions (1.9%), of which 15 were landraces from India, Afghanistan, Nepal, and 7 were from Ethiopia. A newly discovered variant, *eIF4E^B^*, was present in 328 accessions (11.7%) from Ethiopia (29%), Afghanistan (23%), India (20%), Israel (25%) and China (39%). The *eIF4E^C^* variant was detected in 91 accessions (3.2% of total) from India (20%), Afghanistan (33%), the Iberian Peninsula (22%) and the Balkans (9.3%). The *eIF4E^S^* variant for susceptibility predominated as the wild type. Sequencing of 73 samples, identified 34 alleles at the whole gene, 26 at cDNA and 19 protein variants, respectively. Fifteen alleles were virologically tested and 9 alleles (*eIF4E^A-1-2-3-4-5-6-7^*, *eIF4E^B-1^*, *eIF4E^C-2^*) conferred resistance to the P1 PSbMV pathotype.

**Conclusions/Significance:**

This work identified novel *eIF4E* alleles within geographically structured pea germplasm and indicated their independent evolution from the susceptible *eIF4E^S1^* allele. Despite high variation present in wild *Pisum* accessions, none of them possessed resistance alleles, supporting a hypothesis of distinct mode of evolution of resistance in wild as opposed to crop species. The Highlands of Central Asia, the northern regions of the Indian subcontinent, Eastern Africa and China were identified as important centers of pea diversity that correspond with the diversity of the pathogen. The series of alleles identified in this study provides the basis to study the co-evolution of potyviruses and the pea host.

## Introduction

Crop genetic diversity is an important pre-requisite for improving crop traits through breeding, particularly since the presence of closely related and genetically uniform varieties provides an ideal genetic environment for disease epidemics to occur, as evidenced by several historical and also recent events (the 1846 potato blight in Ireland, the 1970 corn blight in the USA, or the 1999 wheat rust in Africa). Available crop genetic resources function as reservoirs of often yet undiscovered allelic variants that provide an opportunity for genetic improvement of a given cultivated species [1], [2]. However, the identification of specific, often rare traits requires specific screening and testing of entire, often very large collections. This is a time- and resource-intensive process. This situation improves, however, once a respective underlying gene is identified, especially in the case of monogenic traits. Various available genomic technologies [3]–[7] can be applied to uncover such variation. Such screening of wild and cultivated germplasm for allelic variation of identified resistance genes has been receiving increased attention [2], [8], as it can be efficiently substituted for phenotypic characterization.

Pea is an ancient legume crop, originating and domesticated in the Middle East and Mediterranean regions, from where its cultivation spread to today's Russia, and westwards through the Danube Valley and/or through ancient Greece and Rome into Europe. Pea likewise spread eastward into Persia, India and China [9]. Archaeological evidence indicates that the first instance of pea dates back to 8000 B.C. [10] in the Near East. Later, during the Stone and Bronze Ages, the existence of pea was documented in Europe and India. From there, it entered China by the first century B.C. [11]. Wild *Pisum sativum* subsp. *elatius* and subsp. *sativum* are found naturally in Europe, Northwestern Asia and in temperate Africa, while *P. fulvum* range is restricted to the Middle East. *Pisum abyssinicum* is found in Ethiopia and Yemen [12], [13]. There are 25 larger sized germplasm collections preserving pea diversity, which collectively hold around 72,000 accessions, while an additional 27,000 accessions are maintained in 146 smaller collections around the world [14], [15]. The diversity of these collections has been studied using both by morphological descriptors and agronomical traits as well as molecular markers (reviewed in Smýkal et al. [14]), and diversity core collections were formed thereafter [16]–[19]. These studies showed that although *Pisum* is a comparably small genus containing two or three species, it has a wide and structured diversity, showing a range of degrees of relatedness that reflect taxonomic identifiers, eco-geography and breeding gene pools [13], [17].

Along with abiotic stresses, plant pathogens are a major detriment to agriculture and threaten global food security. The use of genetic resistance is considered to be the most effective and sustainable strategy for controlling plant pathogens in agricultural practice, as it is environmentally friendly, targets specific pathogens, and provides reliable protection without additional labor or material costs [20]. Long before plants were domesticated and grown as monocultures, plant pathogens were co-evolving with wild plants growing in mixed-species communities. Evolution has continued to occur within domesticated plants growing as selected genotypes in denser populations than in the wild. Furthermore, the domestication of wild plants has distributed crops far from their places of origin [21], [22], and the introduction of pathogens eventually accompanied this distribution. This co-evolutionary process shaped both plants and their pathogens, including viruses [23]–[25].

*Pea seed borne mosaic virus* (PSbMV), member of the genus *Potyvirus*, has been since identification in Czechoslovakia [26] reported worldwide and causes serious yield losses in a broad spectra of legumes including the most economically important like pea, lentil, faba bean, and chickpea [27]. The virus causes various symptoms, depending on the host and virus isolate/pathotype, such as the downward rolling of leaflets, the transient clearing and swelling of leaf veins, chlorotic mosaics, stunting, and delayed flowering. PSbMV is transmitted between plants in a non-persistent manner by aphids and then infects seeds [28]. Due to its seed-borne transmission, PSbMV presents a serious phytosanitary risk both for germplasm maintenance [29] and seed production.

The pea genome contains two virologicaly defined clusters of recessive resistance genes that are responsive to various potyviruses. One cluster (on pea linkage group II) includes *bcm, cyv-1, mo, sbm-2* and *sbm-3* loci, conferring resistance to *Bean common mosaic virus* (BCMV) *Clover yellow vein virus* (ClYVV), *Bean yellow mosaic virus* (BYMV-S s pathotype) and *Pea seed-borne mosaic virus* (PSbMV, pathotype P2) respectively. The second cluster (on pea linkage group VI) includes *cyv-2, wlv* and *sbm-1* loci, conferring resistance to *Clover yellow vein virus* (ClYVV), *Bean yellow mosaic virus* (BYMV-W pathotype) and the *Pea seed-borne mosaic virus* (PSbMV, pathotype P1), respectively [30], [31]. With exception of *cyv-2* and *sbm-1* loci, shown to be identical [32], [33], it is not clear if these clusters are closely linked separate genes or the same gene with alleles of different specificity.

The pea *eIF4E* gene was identified as a susceptibility factor corresponding to the recessive resistance gene, *sbm-1* locus, and a homologue *eIF(iso)4E* is presumed to be *sbm-2* locus [31]–[35]. PSbMV has been well-studied genomically [36]–[38] and viral P3-6K1 and VPg proteins have been identified as PSbMV determinants [30] responsible for physical interaction with host eIF4E or eIF(iso)4E proteins and critical for viral infection. Studies on pepper and *Arabidopsis* suggest that potyviruses may selectively use either eIF4E and/or eIF(iso)4E proteins to achieve infection [39], [40].

Recessive resistance of pea to PSbMV corresponds with the matching-allele model, explaining the interaction between potyviruses and plant hosts [41]. The mutant resistant allele of pea *eIF4E* (named *eIF4E^A^* in this study) differs from its wild-type (sensitive) counterpart by five non-conservative amino acids [32], [31], [42]. Natural variation and functional analysis have revealed evidence of co-evolution between *eIF4E* and potyviral VPg [3], [43], [44]. The *eIF4E* allelic diversity has been systematically screened in various crop collections, such as pepper [5], [45], [46], melon [4], tomato [47] and barley [3]. A possible link between the spread of potyviruses and the origin of agriculture was demonstrated by Gibbs et al. [48], who clearly showed that the human-mediated spread of crop hosts was followed by the further diversification of viruses.

In this study, we have combined our knowledge of pea germplasm diversity with that of the *eIF4E* resistance gene and systematically screened 2803 accessions with known geographical origins including *Pisum* species with the aim of identifying allelic diversity present within the broader pea germplasm held in *ex situ* collections.

## Results

### Geographical distribution of eIF4E variants

**Figure 1:**
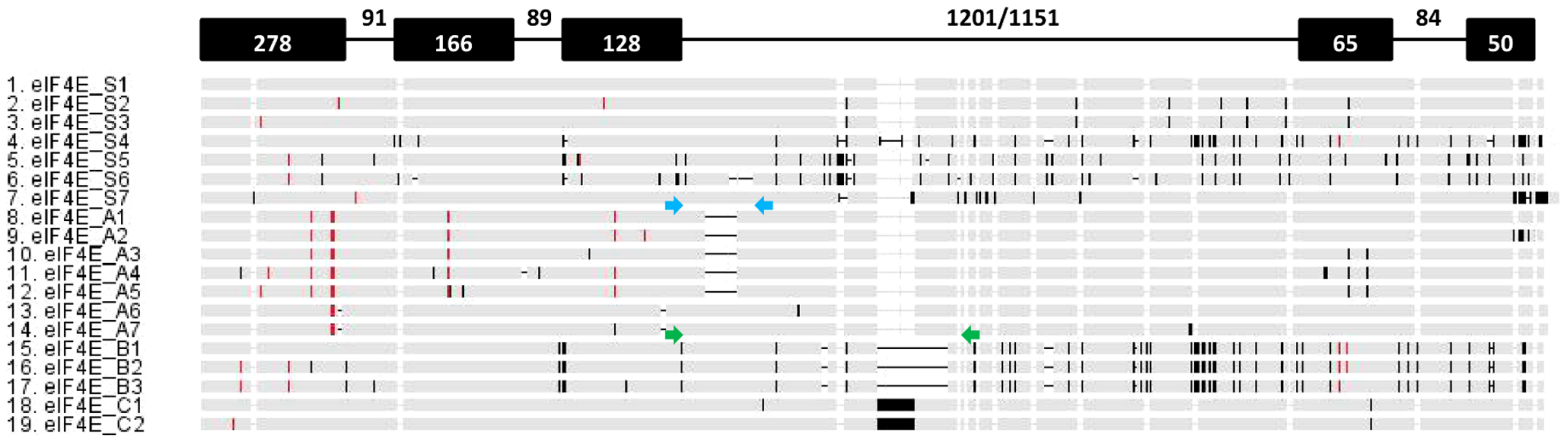
Schematic representation of sequence alignment of the all 19 identified protein *eIF4E* alleles. Four principal *eIF4E* variants identified by intron 3 polymorphism are designated A,B,C and S, while numbers indicate the respective allelic variant. Black bars indicate polymorphism nucleotides within both exons and introns, while red bars indicate polymorphism leading to amino acid exchanges. Horizontal lines indicate insertions/deletions. The heading line indicates nucleotide numbers and exon (solid black boxes)-intron (lines) positions and sizes. Blue arrows indicate primer A combination (Ps-eIF4E-750F and Ps-eIF4E-586gR) and green arrows indicate primer B combination (Ps-eIF4E-750F and Ps-eIF4E-1270R).

**Figure 2:**
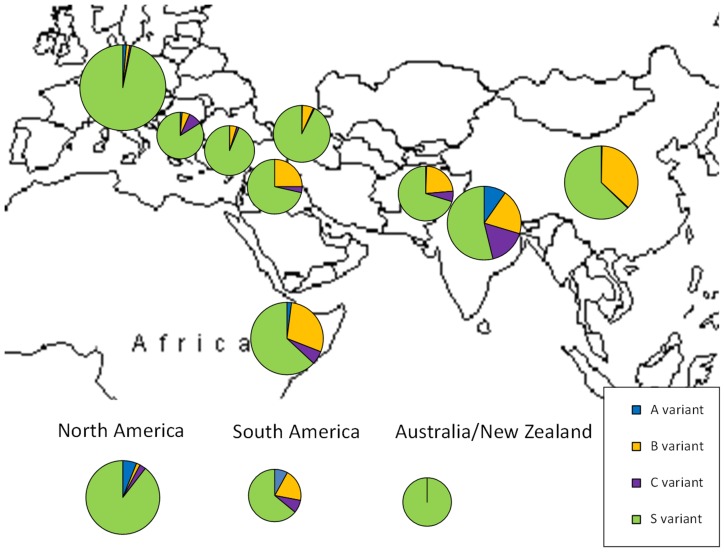
Geographical distribution of four *eIF4E* alleles expressed as percentage of total. Comparison of allele frequencies in 13 geographical regions, as detailed in **Table 2**.

A total of 2803 pea accessions were screened for *eIF4E* intron 3 length polymorphism using two sets of primer combinations, resulting in the detection of four respective *eIF4E^A-B-C-S^* variants (Table 1, Fig. 1). The first corresponded to the already known *eIF4E^A^* resistance [31] variant (amplified 243 bp fragment with primer combination A and 536 bp with primer combination B), the second to the susceptible *eIF4E^S^* [31] (293 and 586 bp fragments), the third to the novel *eIF4E^B^* (293 and 536 bp fragments) and the fourth to the novel *eIF4E^C^* (293 and 592 bp fragments). The resistance *eIF4E^A^* variant was found in seven accessions from Ethiopia-Sudan, fourteen accessions from India, five accessions from Pakistan-Nepal and one accession each from China, Russia and Afghanistan. In addition, this variant was detected in fifteen USA and Canadian, two South American and eleven European modern varieties or breeding lines, which have the Ethiopian line PI193835 in their pedigree. In total, the *eIF4E^A^* variant was detected in 53 accessions, which represent 1.9% of those tested. The *eIF4E^B^* variant was abundant in accessions of Ethiopian (29%), Afghan (23%), Nepal-Indian (20%), Caucasus (6.4%) and particularly Chinese (36.5%) origin (Table 2, Fig. 2). Moreover, it was found in five (e.g. 20%) accessions of South American origin. In contrast, this variant was under-represented in landraces of European origin, except in the Balkans (4 acc.), and absent from modern pea varieties. The *eIF4E^C^* variant was the most frequent in Iberian peninsula (25%) and India-Nepal (16.6%), followed by Balkan (9%), Ethiopia (6%), and Afghan (6.2%) locations, and occurring in total of 91 (3.2%) accessions (Table 2). Due to bulking of 10 plants per sample in the case of Chinese origin accessions (ATFCC), we detected high proportion (15%) of sample heterogeneity. This heterogeneity was not possible to test in USDA, IPK, CGN and JIC samples, as they originated from single plants, while in CzNPC and VIR samples (originated again from bulks of 10 plants per samples) prevailed susceptible alleles. Finally, the susceptible *eIF4E^S^* variant was found in 53.8% to 100% proportion within studied regions, in total 2331 accessions (83%). The lowest occurrence was in 145 studied accessions from India (53.8%), followed by China (63%) and Ethiopia (63%). In contrast, within the 1145 analyzed European origin accessions, including modern pea varieties, it was predominant (97%) (Fig. 2, Table 2). It is clear that there is bias towards European (1145 acc.) compare to other geographical regions, affecting likely allele distribution resulting in S allele over-representation.

**Table 1:** Allele designation based on intron 3 length polymorphism, indicating size of obtained fragments with primer combinations A and B, number of repeat motives, intron 3 difference and total length.

*eIF4E variant*	Primer combination A product (bp)	Primer combination B product (bp)	copies of 50 bp repeat	copies of 56 bp repeat	intron 3 difference	intron 3 length (bp)
**S**	293	586	2	2	0	1201
**A**	243	586	1	2	−50 bp	1151
**B**	293	536	2	1	−70 bp	1131
**C**	293	592	2	3	+56 bp	1257

**Table 2:** Summary of germplasm screening of 2803 pea accessions, indicating four principal *eIF4E* intron 3 length variants (A-B-C and S), distribution in total numbers and percentage over 13 geographical regions.

Regions						Percentage				
	Number of accesions	A variant	B variant	C variant	S variant		A variant	B variant	C variant	S variant
**Ethiopia - East Africa**	349	7	101	21	220		2.0	28.9	6.0	63.0
**Afghanistan-Pakistan**	178	1	41	11	125		0.6	23.0	6.2	70.2
**India-Nepal**	145	14	29	24	78		9.7	20.0	16.6	53.8
**China-Mongolia**	271	1	99	1	170		0.4	36.5	0.4	62.7
**Turkey-Syria**	64	0	3	1	60		0.0	4.7	1.6	93.8
**Israel-Lebanon-Jordan**	28	0	7	1	20		0.0	25.0	3.6	71.4
**Balkan**	75	1	4	7	63		1.3	5.3	9.3	84.0
**Russia-Caucassus**	295	1	19	2	273		0.3	6.4	0.7	92.5
**Iberian Peninsula**	36	0	2	9	25		0.0	5.6	25.0	69.4
**Australia-New Zealand**	11	0	0	0	11		0.0	0.0	0.0	100.0
**South America**	25	2	5	2	16		8.0	20.0	8.0	64.0
**Europe**	1145	15	15	7	1108		1.3	1.3	0.6	96.8
**USA-Canada**	181	11	3	5	162		6.1	1.7	2.8	89.5
**Total**	**2803**	**53**	**328**	**91**	**2331**	**Average**	**1.9**	**11.7**	**3.2**	**83.2**

### Isolation of eIF4E sequences from the selected accessions

In order to assess the genetic diversity within the four classes (A, B, C and S) and to test whether intron 3 lengths correspond to DNA and amino acid haplotypes; we sequenced 32, 25, 9 and 7 accessions with respective *eIF4E^A-B-C-S^* variants from selected geographically diverse regions: Turkey, Nepal, Pakistan, Afghanistan, Ethiopia and China (Fig. 2, Table 2, 3, Table S2). The total length of genomic DNA clones of *eIF4E^A-B-C-S^* variants from ATG to stop codons was 2102, 2080, 2208, 1973–2892 bp long, respectively (Table S2), and length polymorphism was largely conferred by 50 and 56 bp insertions/deletions of minisatellite-like repeat sequences located at 102 and 421 bp respectively from the beginning of intron 3 (Fig. 1, Table 1). The extensive variation of *eIF4E^ S^* was due to wild *Pisum* samples, especially of more distant subspecies (*P. abyssinicum*) and species (*P. fulvum*). Beside intron 3 polymorphism, extensive nucleotide variation was found; altogether, there were 156 SNPs identified in 73 sequences (Fig. 1, Table S2) resulting in 34 alleles at whole gene (exones-intrones), 26 at cDNA and 19 alleles at protein levels, respectively. Of these 156 SNPs, 62 were unique, e.g. occurred only in once within the sequenced samples.

**Table 3:** List of sequenced 73 accessions assigned to identified 19 protein alleles of four *eIF4E ^A-B-C-S^* intron 3 length variants with indicated country of origin.

*eIF4E* allele	Number of accessions with given allele	Accession(s)	Origin
***eIF4E^S-1^***	24	JI 182, JI 1785, JI 1030, JI 190, PI 357290, JI 193, JI 194, JI 205, JI 1107, JI 1085, JI 2065, JI 1845, JI 1532, JI 2607, JI 1121, JI 1756, JI 2571, JI 3001, JI 2630, JI 1091, JI 3157, JI 261, ATC_7173, ATC_6927	Nepal, Nepal, Iran, Sudan, Macedonia, Sudan, Sudan, Russia, Nepal, Turkey, Tanzania, Greece, Hindukush, Lybia, Nepal, Nepal, Gruzie, Iran, Crimea, Greece, Turkey, Turkey, China, China
***eIF4E^S-2^***	1	PI 347328	India
***eIF4E^S-3^***	3	PI 347422, PI 347484, ATC_6931	India, India, China
***eIF4E^S-4^***	1	JI 1632	Ethiopia
***eIF4E^S-5^***	1	JI 1007	Israel
***eIF4E^S-6^***	1	JI 1010	Israel
***eIF4E^S-7^***	1	JI 2646	Malawi
***eIF4E^A-1^***	19	JI 967, JI 467, JI 1790, ATC_1044, PIS_468, PIS_479,CGN_3302, PI 116056, PI 193584,PI 249645, PI 356991, PI 356992, PI 347494, PI 347492, JI 1260, PI 193586, JI 2643, JI 1787, JI 1788	Ethiopia, Europe, Afghanistan, Afghanistan, Afghanistan, Ethiopia, Ethiopia, India, Ethiopia, India, India, India, India, India, India, Ethiopia, Malawi, Nepal, Nepal
***eIF4E^A-2^***	1	JI 1546	Ethiopia
***eIF4E^A-3^***	1	PI 193835	Ethiopia
***eIF4E^A-4^***	1	PI 378158	India
***eIF4E^A-5^***	1	PI 347464	India
***eIF4E^A-6^***	1	PI 269818	England
***eIF4E^A-7^***	1	PI 269774	England
***eIF4E^B-1^***	7	ATC_7134, CGN_3311, CGN_3319, ATC_7140, PIS_477, JI 1194, ATC_3275	China, Pakistan, Pakistan, China, Hindukush-Pakistan, Afghanistan, China
***eIF4E^B-2^***	1	JI 1370	Turkey
***eIF4E^B-3^***	1	JI 1090	Turkey
***eIF4E^C-1^***	6	JI 267, PI 505122, PI 639981, JI1109, JI1104, JI1108	Greece, Albania, Bulgaria, Nepal, Nepal, Nepal
***eIF4E^C-2^***	1	VIR 1589	Russia, Sverdlovsk

**Figure 3:**
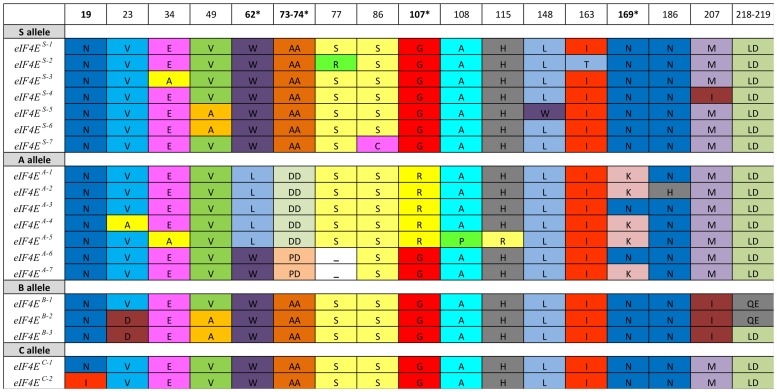
Summarized amino acid exchanges of identified 19 *eIF4E* alleles. Asterix indicates the position of exchanges previously identified as crucial for resistance.

There were four different sequences of susceptible *eIF4E^S^* found within 25 sequenced samples of the cultivated *Pisum sativum* gene pool and three additional sequences within 7 samples of wild pea species (Table 3, Table S2). Translation of computationally spliced cDNA has resulted in four (*eIF4E^S-1-2-3 and 7^*) protein variants within the cultivated genepool and an additional three (*eIF4E^S-4-5-6^*) in *P. fulvum* and *P. abyssinicum* (Fig. 3, Table S2). The typically resistant *eIF4E^A-1^* allele [31], [39], as represented by the PI193835 accession from Ethiopia (genebank sequence number GU289735), differed from the *eIF4E^S-1^* allele (represented by JI194, genebank sequence number KF053441) by five nucleotide (G185T, C218A, C221A in exon 1, G410A in exon 2 and T687G in exon 3) exchanges, while the *eIF4E^A-6^* allele (PI269818 accession, genebank sequence number KF053455) differed by an additional two nucleotide (G217C in exon 1, C993T in intron 3) exchanges, one AGC triplet (AGC229-231) deletion, and one single base (C768T) deletion, in addition to 1200 bp intron 3 length similar to susceptible 1201 bp (Table 2, Table S2). Within the 28 sequenced accessions with *eIF4E^B^* intron length variant, we detected seven alleles, both at nucleotide (Fig. 1) and amino acid (Fig. 3) levels. The three *eIF4E^B-1-2-3^* alleles found in nine sequenced samples differed from the *eIF4E^S^* alleles in 40 SNPs and one nucleotide insertion, resulting in four DNA and three protein variants. The deduced amino acid sequence of the *eIF4E^B-1^* allele differed from the susceptible *eIF4E^S^* allele at three amino acid (M207I and LD218-219QE) exchanges in exons 4 and 5. Two accessions from Turkey (JI1370, JI1090) differed by an additional three SNPs in exon 1, leading to two amino acid exchanges, V23D and V49A, resulting in two *eIF4E^B-2-3^* alleles (Table 3). We have detected two *eIF4E^C^* alleles among seven sequenced samples. The *eIF4E^C-^* ^2^ allele of the VIR1859 accession, besides having a 1257bp long intron 3 due to its extra copy of a second 56 bp repeat, differed by two (A56T in exon 1, G1872A in intron 3) nucleotides from the *eIF4E^S^* allele, leading to single amino acid (N19I) exchange at exon 1 (Fig. 3). The remaining six sequences of *eIF4E^C^* were identical to the *eIF4E^S^* allele, except for a 56 bp insertion in intron 3 and a single nucleotide exchange at intron 3 in PI505122 and PI639981, accessions from Albania and Bulgaria (Table 2, S2). We were interested in establishing which, if any, of the *eIF4E A, B* or *C* alleles can be found in wild pea. It turned out that all analyzed wild pea accesions displayed the *eIF4E^S^* variant as assessed by intron 3 length. Similarly, sequencing analysis of the seven selected accessions had shown that all *P. sativum* subsp. *elatius*, except for *P. fulvum* JI1007, JI1010 and *P. abyssinicum* JI1632 accessions with one or two amino acid exchanges, had the typically susceptible *eIF4E^S-1^* allele (Table 2, 3, S2). All had substantial sequence polymorphism compare to cultivated pea except for *P. sativum* subsp. *elatius* JI2630 from the Crimea.

### Haplotype network analysis

**Figure 4:**
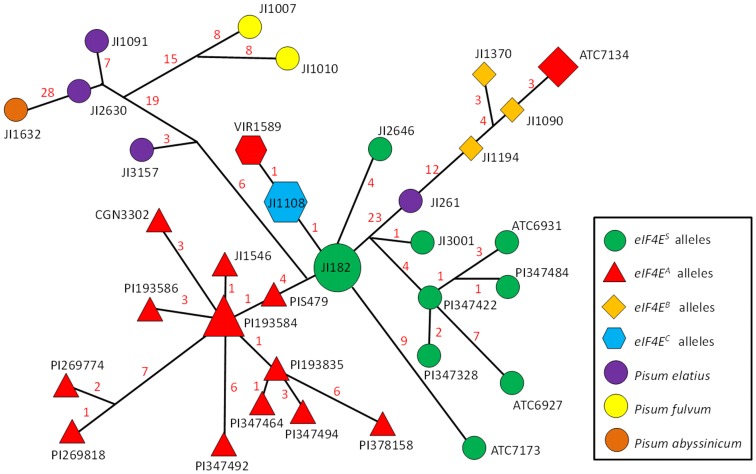
Haplotype network of 34 alleles identified at *eIF4E* whole gene level, using median-joining network algorithm, implemented in NETWORK. It is based on total of 156 SNP characters, and excludes 50, 56Table 1. Red colour of symbols indicates accessions tested resistant to P1 PSbMV. Size of symbols is proportional to number of accessions with given haplotype.

Haplotype network analysis was used to reveal relationships between sequences. This showed the clear separation of *Pisum sativum* subsp. *elatius* (JI3157, JI1091, JI2630), *Pisum fulvum* (JI1007, JI1010) and *Pisum abyssinicum* (JI1632) accessions from cultivated germplasm (Fig. 4). The only exception was *Pisum sativum* subsp. *elatius* JI261 (genebank sequence number KF053440) from Turkey, which had a sensitive allele at the amino acid level, while exhibiting a transitory stage towards the *eIF4E^B^* allele at the nucleotide level (Table S2). Moreover, intron 3 was 36 bp longer, with a length of 1187 bp. Interestingly, the JI261 sequence was identical to *eIF4E^S^* until position 956 of intron 3, from which point forward it was similar to the *eIF4E^B^* allele. An important difference was the absence of the exon 4 and 5 exchanges, where it was again identical to the susceptible *eIF4E^S^* allele. The JI261 sequence occurred between the *eIF4E^S^* and *eIF4E^B^* alleles, with 23 mutational steps. A further 24 mutations separated the *eIF4E^B^* alleles of JI1194 (genebank sequence number KF053439) of Afghanistan, ATC7134 (KF053433) of China, and JI1090 (KF053434) and JI1370 (KF053432) of Turkey (Fig. 4). The *eIF4E^A^* alleles identified in Indian accessions (PI347464, PI347494, PI378158) proved to be derived from Ethiopian (PI193835, PIS479), Afghan (PIS468) and Indian (PI356991, ATC1044) accessions, and were identical. A further cluster contained Chinese (ATC6931, ATC6927) and Indian (PI347484, PI347422 and PI347328) origin S alleles, both at nucleotide and amino acid levels (Fig. 4).

### Testing for response to PSbMV infection

The functional status of the 15 alleles of all four detected variants was verified through biological testing of their response to P1 PSbMV infection. All 19 of the pea accessions bearing *eIF4E^A-1^* tested showed resistance to viral infection i.e. the same phenotype as the non-infected or resistant controls (Table S2). DAS-ELISA testing proved negative, indicating the absence of viral coat protein. Six samples had *eIF4E^A-2-3-4-5-6-7^* alleles with single (PI116056, PI193584, JI1788) or multiple (PI269818, PI269774) amino acid exchanges, but none of these changed the expression of resistance in these accessions. The eight tested accessions bearing the *eIF4E^B-1^* allele split into two phenotypic groups. Three accessions (ATC7134 from China and CGN3311, PIS477 from Pakistan, all *eIF4E^B-1^*) were resistant to the P1 pathotype of PSbMV, as the virus was not able to replicate and spread. The second group of accessions (ATC7140, ATC6928 from China, CGN3319 from Pakistan, all *eIF4E^B-1^*; and JI1090, *eIF4E^B-2^*, from Turkey), showed the typical susceptibility symptoms of infection, vein clearing and chlorotic leaf mosaics, leaf size decrease, internode shortening, and sporadically mild leaf-rolling, 14 days after inoculation by PSbMV. The symptoms were comparable with the response of the sensitive genotypes with the *eIF4E^S-1^* allele. All tested *eIF4E^S-1^* (18 accesions), *eIF4E^S-2^* (1 acc.) and *eIF4E^S-3^* (3 acc.) displayed sensitive response to P1 PSbMV pathotype (Table S2). We have not been able to test remaining *eIF4E^S-4,5,6^* ^and 7^ alleles, due to the shortage of homogenous plant material. Finally, of the three tested accessions with the *eIF4E^C-1-2^* alleles, only VIR1589 from the Sverdlovsk region of Russia was resistant, while JI1104 and JI1108 from Nepal proved susceptible (Table S2). This corresponded to an amino acid exchange at position N19I in exon 1 between the *eIF4E^C-1^* and *eIF4E^C-2^* alleles (Table S2).

## Discussion

In this study, we combined our knowledge and access to a wide range of pea germplasm [13], [17] with an interest in PSbMV resistance [31] and focused our analysis on the geographical distribution of selected, easily detectable alleles of the *eIF4E* gene using the polymorphism of intron 3.

### eIF4E gene structure and diversity

The pea *eIF4E* gene is comprised of five exons, interrupted by four intron sequences, with a total length of 2.1 kb [31], which is spliced into a 687 nucleotide long (open reading frame) mRNA [32]. The size polymorphism of intron 3 is caused by 50 and 56 bp insertions of minisatellite-like repeat sequences [31], separated by 259 bp of common intron sequence. This polymorphism led to the initial mapping of the *sbm-1* locus [35] and the development of a co-dominant marker for breeding [31]. Previous sequencing of 43 accessions of pea varieties showed four haplotypes of intron 1, eight haplotypes of the intron 2 and three haplotypes of intron 4, while 17 haplotypes were detected in intron 3 sequences ([31] and unpublished). Since all, except PI269774 (Sankia) and PI269818 (Aa134), previously tested susceptible and resistant accessions differed by 50 or 56 bp indels in intron 3 [31], we exploited this length polymorphism to screen a wider pea germplasm population. This led to the identification of novel polymorphisms in four principal variants (*eIF4E^S^*, *eIF4E^A-B-C^*). Another functional *eIF4E* resistance allele found in the PI269818 and PI269774 accessions was not directly tested in this study, as its polymorphism is more difficult to analyze. It is clear that this type of analysis does not provide a comprehensive dataset, as more subtle, single-nucleotide mutations are missed, as documented by sequenced samples, whereas in 73 sequences, 19 *eIF4E* alleles were found (Fig. 1, Table 3, S1).

The mutant *eIF4E^A-1^* allele of pea gene *eIF4E* differs from its *eIF4E^S-1^* counterpart in five non-conservative amino acid exchanges in and around the cap-binding pocket of pea *eIF4E*, which impacts infection by PSbMV [32]. Previously reported mutations in pea *eIF4E* were localized on the β1 (W62L), β1-β2 (AA73-74DD), β3-β4 (G107R) and β5 (N169K) loops [42]. Positions of the newly identified amino acid (V23D, V49A and LD218-219QE) changes in *eIF4E^B-1-2-3^* alleles do not correspond with the known mutations leading to resistance and are not localized on the variable β loops regions, with the exception of the M207I change located on the α3-β7 loop. The only resistance determinant mapped to the β7 loop is for BaYMV infection in barley [54]. In the *Capsicum* species, several *eIF4E* haplotypes with amino acid changes were reported, some of which included amino acid changes in positions 218 and 219 similar to those in the pea *eIF4E^B^* allele. Systematic testing of individual mutations by *in planta* assay showed that only a specific combination of mutations leads to resistance [42]. This hypothesis confirms previously reported amino acid changes in pea eIF4E in PI269818 and PI269774 accessions [31] with *eIF4E^A6-7^* alleles, which have amino acid changes in the cap-binding pocket [31] and lack one (S78) amino acid (Fig. 3, Table 3 and Table S2, Supporting information). Amino acid changes in the loop near the cap-recognition pocket were shown to be directly associated with resistance to potyviruses in pepper, lettuce, and pea [35], [55]. Moreover, not all *eIF4E* proteins encoded by the resistance alleles are defective in their ability to bind the m7-GTP cap, suggesting that disrupted cap binding is not always required for potyvirus resistance [56]. Thus mutations conferring *sbm-1* resistance to PSbMV act combinatorially [42]. The interactions between particular *eIF4E* and particular viral VPgs are highly specific for host-virus interactions. Higher plants are unique in that they encode two distinct isoforms of eIF4F that have both overlapping and isoform-specific roles, eIF4F protein complex, which contains eIF4E and eIF4G, and eIF(iso)4F protein complex, which contains eIF(iso)4E and eIF(iso)4G [25]. Although these two complexes seem to be equivalent for the *in vitro* translation, they differ in their *in vivo* expression patterns and specificity for cellular mRNAs and likely also viral RNA. Potyviruses could selectively use either *eIF(iso)4E* or *eIF4E* to infect plants, while some are able to use both [39]. In pepper, the combination of *eIF4E* (*pvr2* locus on chromosome 4) and *eIF(iso)4E* (*pvr6* locus on chromosome 3) resistance alleles showed a complementary effect [45]. Also in pea, the *eIF(iso)4E* homologous gene exists, mapped to LG II ([35] and close to the *sbm-2* locus (not shown). However, its direct involvement in potyvirus resistance still needs to be demonstrated. Thus we cannot exclude the possibility that interaction between eIF4E and eIF(iso)4E proteins may also play a role in pea. This might partly explain the discrepancies in virological testing of *eIF4E^B^* accessions. Finally, the result of the interaction, the resistance or tolerance, depends not only on the genetic background but also on the virus pathotype [43], [57]. It would be interesting to test region-specific potyvirus isolates [58] on the alleles identified in this study. The current phylogeny of potyviruses indicates that the progenitor probably infected plants growing in southwestern Eurasia and northern Africa, which evolved from a rymovirus by acquiring the ability to be transmitted by aphids [48]. It is notable that we did not find the resistant *eIF4E^A^* variant in any of the 146 tested wild pea samples, nor have we been able to detect any intermediate alleles between the susceptible *eIF4E^S-1^* and resistant *eIF4E^A-1^*, except in JI261 accessions already separated by 23 mutations between *eIF4E^S-1^* and *eIF4E^B-1^.* Such an intermediate allele was found for *Pm3* powdery mildew resistance in wheat from the Himalayan Range, supporting the hypothesis that a recent evolution of these alleles took place in the hexaploid wheat gene pool [8]. This is a departure from the situation with other diseases for which resistance can often be found within the wild genepool, in contrast to the domestic gene pool. i.e. pathogens overcame resistance in the domestic gene pool. Haplotype analysis indicated the separate evolution of *eIF4E^A^* alleles, with Ethiopian and Indian accessions being the most distantly related, while Chinese and some Afghan and Nepalese accessions were closer to *eIF4E^S-1^* (Fig. 4). Thus the evolution of the *eIF4E* gene is demonstrated in the available pea genepool.

### Geographical distribution of eIF4E variants versus PSbMV diversity

**Figure 5:**
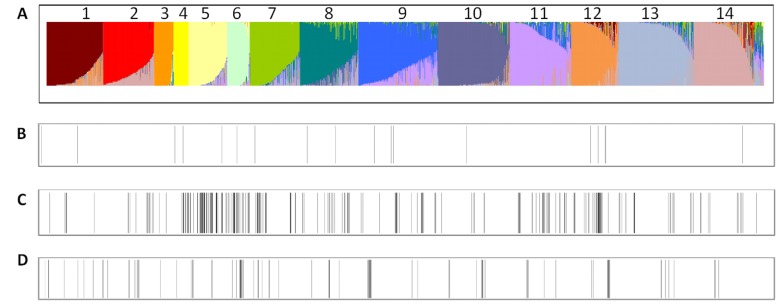
Visualization of genetic relationship of four identified *eIF4E* variants on the background of 14 BAPS identified clusters of 3,029 JIC accessions genotyped by 45 retrotransposon-insertion loci [13], [63]. Notably, clusters 3 and 4 contain 145 acc. of wild *P. fulvum, P. s.* subsp. *elatius* and *P. abyssinicum*; cluster 7 contains 95 acc. from Afghanistan; cluster 8 has 225 acc. from Ethiopia; and cluster 9 contains 247 acc. from India and Ethiopia, while remaining clusters are more diverse (A). 18 accessions with the *eIF4E^A^* (B), 241 accessions with the *eIF4E^B^* (C), 81 accessions with the *eIF4E^C^* variant (D).

Pea genotypes resistant to PSbMV were identified by Hagedorn and Gritton [59] in two Ethiopian lines (PI193586 and PI193835). Later, Hampton [60] found several resistant accessions from India. Since they are all identical in sequence [31], it raises the question of their origin. Hampton [60] proposed that India may have been the center of origin for the virus, and consequently also the center of PSbMV-immune germplasm. Despite this resistant germplasm, pea has not been systematically bred to include PSbMV resistance until recently; as a consequence, the vast majority of pea varieties are susceptible to this virus [31], especially when contrasted with landraces where the possibility of both natural and human selection for resistance exists. A relationship between pea host and PSbMV pathogen diversity in northern Pakistan and Afghanistan has been proposed [60], [61]. More recently, phylogenetic analysis of PSbMV isolates from Australia and China grouped them on separate clades [55]. These results suggest the operation of co-evolutionary forces, which could be tested using the *eIF4E* alleles identified in this study. Interestingly, similar results were found in a study of barley *eIF4E* haplotype diversity, which was also found to be considerably higher in Central and East Asia, both regions with a long history of the bymovirus disease [3]. It is intriguing to see that in different crops, the resistance diversity center is outside the original place of domestication, especially in Central and Eastern Asia. It might be hypothesized that only some environments may be conducive for pathogen development in crops, hence co-evolution may be geographically limited. This agrees with results showing that the *eIF4E* gene is under positive selection pressure [3], [44]. There is strong selection for the *eIF4E^B^* allele in China and less elsewhere (Fig. 2, Table 2), but no A or C alleles in China, possibly consistent with a genetic bottleneck in the later migration of pea to China, compared with central/western Asia [18]. Moreover, assignment of Chinese accessions to provinces showed preferential occurrence of B alleles in the autumn/winter-sown provinces of Henan, Anhui, Hubei and Sichuan (Table S1) that experience high rainfall, long growing seasons and cold, frost-prone vegetative phases. Conversely, S alleles predominated in spring-sown types grown in northern provinces with low rainfall and high, variable temperatures that result in severe drought [62]. Analysis of pea germplasm diversity, using retrotransposon-based insertion polymorphism [13], [14], [63] showed that although there is substantial genetic diversity present, it is only partially geographically structured. Especially in case of the *eIF4E^B^* alleles, there is substantial clustering of Indian and Ethiopian accessions, indicating a close relationship between materials from these areas (Fig. 5). The number of mutations makes independent origin unlikely, unless stringent positive selection is operating, as detected by Cavatorta [44]. We have also found evidence of positive selection in a set of 27 tested pea cDNA haplotypes (not shown). As proposed for barley *eIF4E*, the explanation for the unusually high overall degree of *eIF4E* sequence variation and haplotype diversity may be that these are important for adaptation to different local habitats [3]. The second, more plausible scenario is introgression and maintenance in populations by selective advantage. Based on the number of accesions with detected *eIF4E^A^* and *eIF4E^B^* alleles in the northern Indian subcontinent versus Ethiopia/eastern Africa, we speculate that these more likely originated in the Africa and were then brought by oversea trade to Afghanistan-Pakistan-Nepal-India region. The Silk Route, Amber Road and trans-Saharan trade routes were all instrumental in establishing links between Africa, India and beyond. This scenario, while not proven, is supported by a haplotype analysis network, where Indian *eIF4E^A^* alleles were shown to be derived from Ethiopian accessions (Fig. 4). There is bias towards European origin accesions, comprising 40.8% of samples, followed by Ethiopia and East Africa (12.4%) and Russia (10.5%) which consequently affects proportion (57%) of S alleles (Table 2). However, these samples were included to provide more complete view on *eIF4E^A^* diversity, including modern pea varieties. However this affects only percentage in total dataset and not within studied regions (Fig. 2, Table 2).

Various approaches have been applied to identify variants of the *eIF4E* gene in several crops. These showed different levels of diversity [3], [43], [46], [47]. In the most comprehensive study, 1090 barley samples from 84 countries screened using the high-resolution melting PCR method of cDNA, led to the identification of 47 *eIF4E* haplotypes [3]. In our study, we have identified 34 alleles on exon-intron level with 156 single-nucleotide polymorphic sites in total of 73 sequences. This suggests that substantial diversity within the *eIF4E* gene can be expected within available pea germplasm, which might be fully revealed only by sequencing. It is notable that none of the 146 tested wild pea samples had *eIF4E^A^* or *eIF4E^B^* alleles, but at the amino acid level, all corresponded to the susceptible *eIF4E^S-1^* allele, despite substantial polymorphism both in introns and exons, indicating selection for functionality. The discovery of the intermediate allele in wild *P. sativum* subsp. *elatius* JI261 from Turkey is similar to data obtained in *Arabidopsis thaliana* [25], which suggests a distinct mode of evolution of resistance in wild species as opposed to crop species and supports the scenario in which potyviruses spread at the advent of agriculture, which brought plants into dense monocultures [48]. Very little is known about the selective forces that drive viral evolution in natural ecosystems, which contrasts with the more detailed population genetics studies in crop plants that have revealed the importance of mutation rates, recombination, genetic drift and migration in virus evolution [64]. Although we can not fully excluded the possibility, that JI261 allele could have resulted from domestic to wild introgression and not independent mutations, the analysis of 45 RBIP loci (Fig. 5) clustered it with typical *P. s.* subsp. *elatius* accessions [63]. The existence of resistance alleles only in the domesticated pea genepool leads us to speculate that the mutation originated during early cultivation. The series of alleles identified in this study provide an excellent basis for testing various potyviruses and pathotypes in which to study the co-evolution of potyviruses and their pea host.

## Materials and Methods

### Plant material

The 2803 pea accessions used in this study were drawn from the following collections: the Czech National Pea Collection (CzNPC, 1252 accessions); the USDA core collection (384); 119 accessions of Chinese origin from the Australian Temperate Field Crops Collection [(ATFCC) now reconstituted as Australian Grains Genebank, (AGG)], the Vavilov Institute (VIR) Russian Federation (69); the Centre for Genetic Resources (CGN), The Netherlands (14); Leibniz Institute of Plant Genetics and Crop Plant Research (IPK), Germany (35); and John Innes Centre (JIC), UK (836). The permission from the relevant institutions was obtained to access the collections, and the respective pea germplasm accessions were donated for this study under Standard Material Transfer Agreement of germplasm resources. Although we specifically targeted local landraces rather than globally grown, modern varieties, the analyses of 1252 accessions from the Czech National Pea collection represent the diversity present in the genepool of cultivated pea in the 19^th^ to 21^st^ centuries [16], [49]. This collection is composed of 972 commercial varieties, 226 breeding lines and 54 landraces, which originate largely from Europe (925), the former Soviet Union (177), and the USA and Canada (77). In contrast, the John Innes Centre pea collection has, in addition to varieties (1071) and breeding lines (61), a large proportion of landraces (600), mutant stocks (585) and wild peas (445). Geographically, this collection is dominated by accessions of Ethiopian (388), Mediterranean (199), Indian (53) and Chinese (37) origin. The Australian ATFCC and USDA pea collections are partially complementary. The former includes the diversity core set of Chinese origin [18], of which 119 were studied here, while the latter has over 6000 accessions, of which a core set of 384 [19] was analyzed in this study. Finally, 69 accessions selected from the VIR collection originated in the Caucasus and Central Asia (Turkmenistan, Kazakhstan, Georgia, and Armenia) as well as in North Africa (Morocco and Algeria). It is worth mentioning that although Ethiopia is known as the origin and occurrence for *P. abyssinicum*, the accessions used in this study were mainly *P. sativum* subsp. *sativum.* This set was complemented with wild *P. fulvum* (9), *P. sativum subsp. elatius* (86), *P. sativum subsp. sativum* (19) (formerly *P. humile/P.syriacum*) (see Smýkal et al. [13] for taxonomical classification) and *P. abyssinicum* (32) (Table S1). These represent both primary and secondary centers of pea diversity as well as primary and secondary gene pools.

### DNA isolation and PCR analysis

Young leaves were harvested from ten (in the case of the Czech, ATFCC, CGN, IPK and VIR collections) or single (in the case of the JIC and USDA collections) randomly chosen plants per accession and stored at −80°C. Genomic DNA was isolated and PCR performed using standard protocols [31]. Products were resolved on 1.5% TBE agarose gel and visualized using ethidium bromide staining under UV-light.

### eIF4E variants amplification

Intron 3 length polymorphism was used as screening criteria, as developed by Smýkal et al. [31]. In short, differences in intron 3 length were assayed using PCR with the following pairs of primers: *Ps*-eIF4E-750F (5′-GGACTAAGAATGCTTCAAATGAAGCTGC-3′) and *Ps*-eIF4E-586gR (5′-GAATCATTTAAGAAGCTCGTGAAGTG-3′) primers (combination A, nested within the combination of B) that amplified 243 bp in the resistant and 293 bp in the susceptible accessions [31]; and (combination B) *Ps*-eIF4E-750F and *Ps*-eIF4E-1270R (5′-ATTCTCGATCACACTAGCCCCCTCC-3′) [35] primers that amplified 536 bp versus 586 bp fragment. The combination of these two assays resulted in the detection of respective *eIF4E^A-B-C-S^* variants (Fig. 1, Table 1). PCR amplification of 1000 bp product from start codon to intron 3 was carried out using primers *Ps*-eIF4E-1F start codon (5′-ATGGTTGTAGAAGACACCCCCAAATC-3′) and *Ps*-eIF4E-586gR. Primers *Ps*-eIF4E-794F (5′-GCTAGATGGTTGTTATGATGTTTATCAG-3′) and *Ps*-eIF4E-2188R stop codon (5′-TTGCTAGTTTGCTACCATGTAAGAACG-3′) were used to amplify a 1500 bp PCR product spanning the rest of *eIF4E* gene from intron 3 to exon 5. The PCR products were purified according to Werle et al. [50] protocol and sequenced using a BigDye Terminator kit (Applied Biosystems, UK) by Macrogene (Amsterdam, The Netherlands).

### Bioinformatics

Primer design and restriction analyses were performed using FastPCR software version 5.1.83 (PrimerDigital Ltd., Finland). The DNA sequences were viewed and edited using Sequence Scanner version 2.0 (Applied Biosystems, UK). CLUSTALW alignment was performed using BioEdit version 7.09.0 [51]. The haplotype alignments were performed using a median-joining network algorithm, implemented in NETWORK 4.5.1.6 [52]. To reveal the genetic relationship of samples, we used previously made Bayesian clustering analysis of the genetic diversity of 3,029 JIC accessions genotyped by 45 retrotransposon-insertion loci [13]. Identified clusters were used as framework to visualize individual *eIF4E* alleles within the set of 836 JIC accessions analyzed in this study. Single nucleotide polymorphism mapping to reference sensitive allele was done using Geneious 6.1.6 analysis software (Biomatters, USA).

### Virological testing

Plants were grown in a substrate Klassman no. 4 (Klasmann-Deilmann GmbH, Germany) in a growth chamber (Microclima 1000, Snijders Scientific, Holland) under a 16/8-h and 22/18°C day/night cycle. Evaluation of resistance/susceptibility to the PSbMV pathotype P-1 was conducted through mechanical inoculation using isolate PSB117CZ [31], [53]. All together, 50 accessions were tested. Ten plants per accession were tested in same block, under the same conditions. Sensitive pea cultivars, Merkur and Raman, were used as a sensitive and B99 as a resistant controls [31]. Symptoms were observed at one-week intervals, and systemic infection was confirmed three weeks after infection with DAS-ELISA (Loewe Biochemica, Germany).

## Supporting Information

Table S1 **List of studied accessions, divided by 7 studied germplasm collection with indicated accession number, name, origin and** ***eIF4E*** **variant.**
(XLSX)

Table S2 **Table of sequenced 73 accessions indicating single-nucleotide polymorphic (SNP) sites in exons and introns (SNP sheet) as well as resulting amino acid exchanges (protein sheet).** Species name, germplasm, country of origin and *eIF4E* allele asignment are shown. Length of all introns and exons is shown in base pairs, with variable intron 3 being highlighted. Results of virological testing with P1 PSbMV are indicated as positive, susceptible (S) and resistant (R) reactions, respectively.
(XLSX)
